# Aspartate β-hydroxylase disrupts mitochondrial DNA stability and function in hepatocellular carcinoma

**DOI:** 10.1038/oncsis.2017.64

**Published:** 2017-07-17

**Authors:** C Tang, Y Hou, H Wang, K Wang, H Xiang, X Wan, Y Xia, J Li, W Wei, S Xu, Z Lei, T M Pawlik, H Wang, M Wu, F Shen

**Affiliations:** 1 Department of Hepatic Surgery, The Eastern Hepatobiliary Surgery Hospital, Second Military Medical University, Shanghai, China; 2 Department of Hepatobiliary Surgery, The Daping Hospital, Third Military Medical University, Chongqing, China; 3 Department of Clinical Database, The Eastern Hepatobiliary Surgery Hospital, Second Military Medical University, Shanghai, China; 4 Department of Surgery, The Ohio State University, Wexner Medical Center, Columbus, OH, USA; 5 National Scientific Center for Liver Cancer, Second Military Medical University, Shanghai, China

## Abstract

The mechanism of aberrant mitochondrial genome and function in hepatocellular carcinoma (HCC) remains largely unknown. Our previous study demonstrated an increased expression of aspartate β-hydroxylase (ASPH) in HCC tissues, which was associated with tumor invasiveness and a worse prognosis. Currently, we unexpectedly observed the presence of ASPH in purified mitochondrial protein fraction. In addition, immunostaining of both exogenously and endogenously expressed ASPH showed a colocalization with mitochondrial biomarkers. This study aimed to investigate whether the mitochondrial ASPH is involved in mitochondrial malfunction in HCC. Our results showed that ASPH overexpression in HCC tissues was correlated with decreased copy numbers of displacement loop (D-loop) and NADH dehydrogenase subunit 1 (ND-1) and enhanced D-loop mutation, suggesting the disrupted mitochondrial DNA (mtDNA) stability. The reduced mtDNA copy numbers were associated with aggressive clinicopathological features of HCC. The loss of mtDNA integrity induced by enforced expression of ASPH was accompanied with mitochondrial dysfunction, which was characterized by the aberrant mitochondrial membrane potential, decreased ATP generation and enhanced reactive oxygen species. In contrast, knocking down ASPH by siRNA in HCC cell lines showed the opposite impact on mtDNA integrity and function. Mass spectrometry and co-immunoprecipitation further identified that ASPH interacted with histone H2A member X (H2AX). ASPH overexpression diminished the interaction between H2AX and mitochondrial transcription factor A (mtTFA), an important DNA-binding protein for mtDNA replication, which then reduced the binding of mtTFA to D-loop region. Collectively, our results demonstrate that ASPH overexpression disrupts the mtDNA integrity through H2AX–mtTFA signal, thereby affecting mitochondrial functions in HCC.

## Introduction

Hepatocellular carcinoma (HCC) is the fifth most common malignancy and second leading cause of cancer-related mortality worldwide.^1^ There are multiple risk factors associated with the development of HCC, including viral hepatitis, aflatoxin exposure and excessive alcohol abuse.^2, 3, 4^ Currently, oxidative stress induced via abnormal mitochondrial regulation has also been proposed as an important factor in the pathogenesis of HCC.^5, 6^

The mitochondria is a cytoplasmic organelle that contributes to generate ATP through oxidative phosphorylation,^7^ produces reactive oxygen species (ROS)^8, 9^ and mediates cell apoptosis signal cascade,^10, 11^ in addition to other cellular functions. Notably, genomic aberrations affecting mitochondrial DNA (mtDNA) have been reported in some human malignancies, including HCC.^12, 13, 14^ The impact of these genomic aberrations on mtDNA has been suggested to enhance tumorigenesis by promoting genomic insults from carcinogens, antagonizing tumor suppressor function, mediating metabolic transition to glycolysis and promoting tumor cell migration.^15, 16, 17, 18^

Interestingly, sequencing of the mitochondrial genomes has revealed somatic point mutations in 52% of tested HCC tissues,^19^ in which the displacement loop (D-loop) region mutational hotspot accounts for 76% of all detected mutations.^20^ Moreover, mtDNA copy number aberrations were frequently identified during HCC development.^12, 13^ Previous studies also demonstrated that over 60% of HCC tissues had a decreased mtDNA copy number when compared with those in non-tumor liver tissues.^12^ The loss of mtDNA copy number was associated with a larger tumor size, presence of cirrhosis and, of critical importance, shorter patient’s survival.^21^ Despite these important observations, the understanding of tumor-specific mechanisms regulating mtDNA alteration in HCC remains limited.

Aspartate β-hydroxylase (ASPH) is a type II membrane protein, with hydroxylase enzymatic activity that adds a hydroxyl group to aspartate or asparagine residue within epidermal growth factor-like domains.^22^ Our own group, as well as other authors, has previously demonstrated that ASPH was one of the most differentially expressed genes in HCC and its overexpression was associated with a reduced survival rate and an increased tumor recurrence rate at 5 years after liver resection.^23, 24, 25^ Although the role of ASPH in promoting tumor cell migration and invasion has been identified, the underlying mechanism remains uncharacterized.^26, 27^ Currently, an unexpected observation of mitochondrial localization of ASPH in immunostaining assay using our prepared antibody specific for the catalytic domain of ASPH led us to assume that ASPH might have a role in mitochondrial function in HCC.

In this study, we characterized the detection of ASPH within the mitochondria of HCC cells, and investigated the impact of expression of ASPH on mtDNA stability and function. The results highlight a previously unreported role of mitochondrial ASPH in HCC development.

## Results

### ASPH localizes on mitochondria in HCC cells and HCC tissues

**Figure 1:**
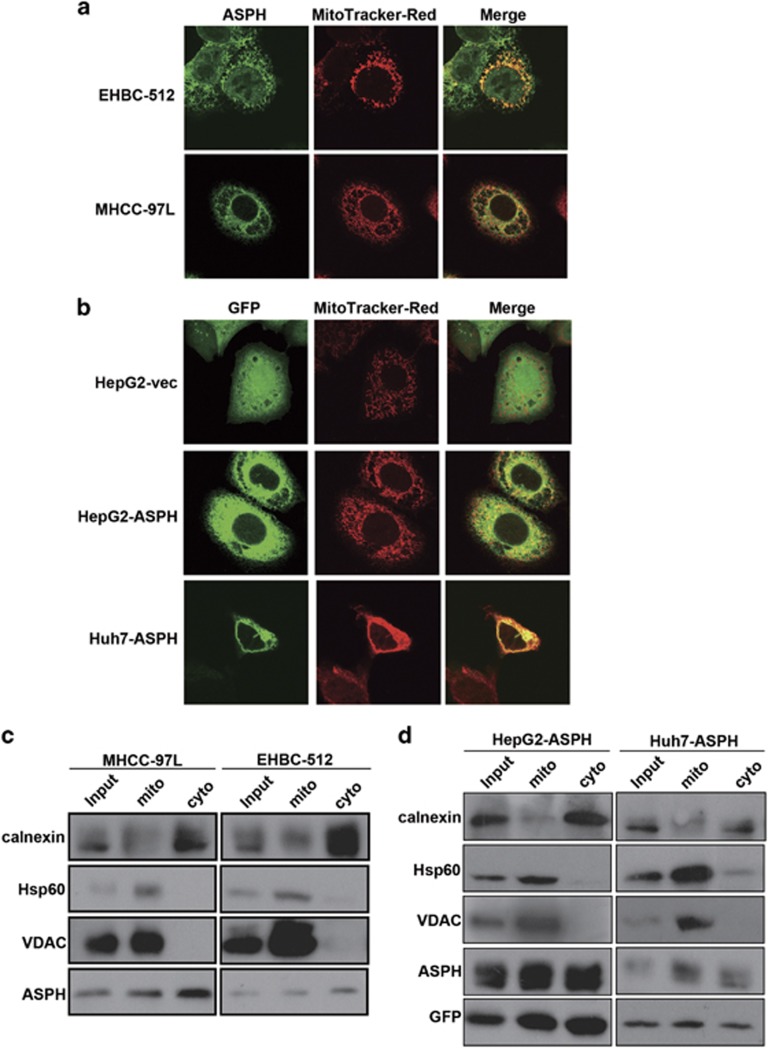
ASPH localizes on mitochondria in HCC cells. (**a**) Colocalization of endogenous ASPH immunostained with anti-ASPH antibodies and mitochondrial marker MitoTracker in MHCC-97 L and EHBC-512 cells. Green: ASPH protein staining; red: mitochondrial organelle staining. Images were taken by confocal fluorescence microscopy under × 600 magnification for oil immersion. (**b**) Colocalization of exogenously expressed green fluorescent protein (GFP)-fusion ASPH immunostained with anti-GFP antibodies and MitoTracker in HepG2 and Huh-7 cells. Images were taken as mentioned above. (**c**) Presence of endogenous ASPH in mitochondrial and cytoplasmic fraction detected by immunoblot in MHCC-97 L and EHBC-512 cells. (**d**) Presence of exogenously expressed GFP-fusion ASPH in mitochondrial and cytoplasmic fraction detected by immunoblot in HepG2 and Huh-7 cells. For **b** and **d**, mito: fraction of mitochondrial protein; cyto: fraction of cytoplasmic proteins. Heat shock protein 60 (HSP60) and voltage-dependent anion channel (VDAC) are biomarkers of mitochondrial organelles. Calnexin is a biomarker of endoplasmic reticulum.

Using an antibody specific against the catalytic domain of ASPH, colocalization between ASPH and the mitochondrial marker MitoTracker was noted in HCC cell lines of EHBC-512 and MHCC-97 L cells, which had a high endogenous expression of ASPH (Figure 1a; Supplementary Figure S1). An overexpression of green fluorescent protein-tagged ASPH was also noted in HepG2 and Huh-7 cells that displayed low endogenous expression of ASPH (Supplementary Figure S1). The green fluorescent protein staining specifically localized on mitochondrial-like structures, featured by the presence of MitoTracker (Figure 1b).

Both endogenous and exogenous ASPH were present in mitochondrial subfraction (Figures 1c and d). These findings were validated by the enrichment of mitochondrial biomarkers heat shock protein 60 and voltage-dependent anion channel. Mitochondrial subfraction of HCC tissues was also isolated. Both endogenous ASPH and mitochondrial biomarker voltage-dependent anion channel were present in mitochondrial subfraction of HCC tissues (Supplementary Figure S2). Moreover, ASPH also had a calnexin-featured cytoplasmic localization (Figures 1c and d). As such, the results collectively demonstrated a mitochondrial subcellular localization of ASPH in HCC cell lines and HCC tissues.

### ASPH expression level is negatively correlated with mtDNA stability in HCC tissues

**Figure 2:**
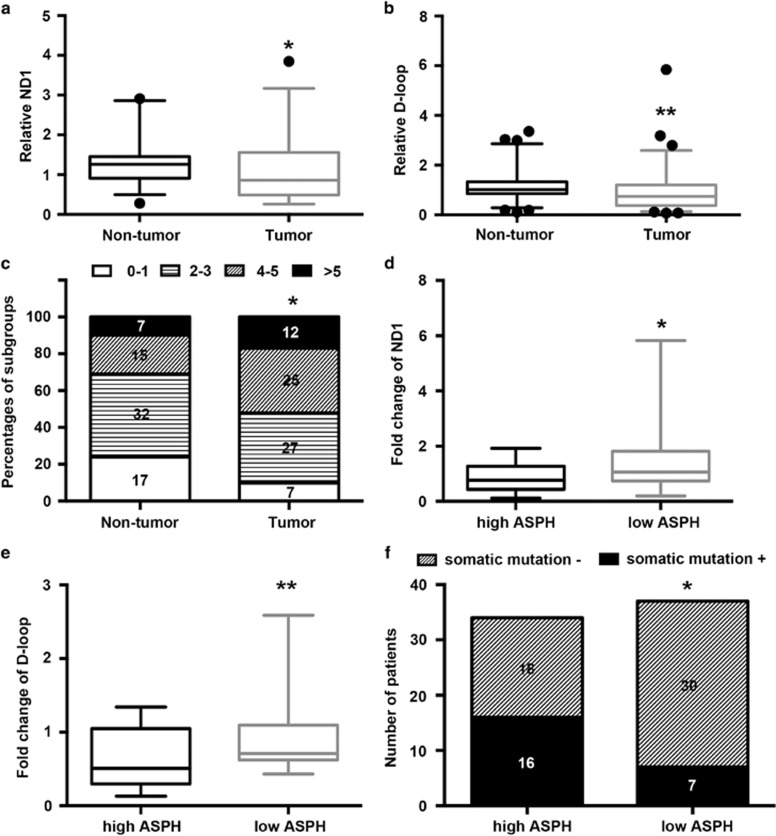
ASPH expression level affects mtDNA stability in HCC tissues. (**a**, **b**) Box plot of the relative copy number of ND-1 and D-loop in mtDNA extracted from tumor and matched non-tumor tissues (*n*=71) that was detected by real-time PCR and normalized to the amounts of β-actin DNA. (**c**) Percentages of subgroups of tumor and non-tumor tissues based on the amount of single-nucleotide variation in the D-loop region identified by Sanger sequencing. The number of patients in each subgroup was marked in the corresponding column. (**d**, **e**) Ratios of tumoral ND-1 and D-loop copy number to matched non-tumor tissues assayed by real-time PCR in the subgroups of HCC patients with high (*n*=34) or low (*n*=37) ASPH expression. (**f**) The number of patients with or without somatic mutation in the D-loop region of mtDNA detected by Sanger sequencing in HCC patients with high (*n*=34) or low (*n*=37) ASPH expression. For all box plots, the band inside the box was the mean value and the ends of the whiskers represented the minimum and maximum of 2.5–97.5 percentile of all data. **P<0.01, *P<0.05 vs groups of non-tumor tissues or high ASPH expression as indicated.

The association between ASPH expression and mtDNA stability based on the changes of mtDNA copy number and mutagenesis was analyzed in tumor and matched non-tumor tissues in a cohort of 71 patients with HCC. A decrease in the copy number status of both NADH dehydrogenase subunit 1 (ND-1) and D-loop was noted in the tumor tissues compared with non-tumor tissues (ND-1: 1.077, 95% confidence interval: 0.904–1.249 vs 1.249, 95% confidence interval 1.133–1.366, *P*<0.05; D-loop: 0.933, 0.727–1.139 vs 1.164, 1.015–1.314, *P*<0.05; Figures 2a and b). Similarly, more mutations were detected in the tumor tissues vs the non-tumor tissues (*P*<0.05; Figure 2c).

HCC tissue samples were further divided into the ND-1-high (*n*=27) and ND-1-low (*n*=44) groups based on the relative ND-1 content in the tumor tissues compared with the matched non-tumor tissues. As shown in Supplementary Table S1, a reduced ND-1 copy number was associated with an elevated serum alpha-fetoprotein level (*P*=0.037), larger tumor diameter (*P*=0.017), presence of microvascular invasion (*P*=0.013) and advanced tumor stage (*P*=0.040). Meanwhile, a reduced D-loop copy number was associated with the absence of tumor capsule (*P*=0.028). Collectively, the data strongly suggested a correlation between the decrease of mtDNA copy number and aggressive pathological features of HCC.

Tumors were then further classified into the ASPH-high (*n*=34) and ASPH-low (*n*=37) groups, as described in the Materials and Methods section. Both ND-1 and D-loop DNA contents were diminished in the ASPH-high group compared with the ASPH-low group (ND-1: 0.863, 95% confidence interval: 0.667–1.059 vs 1.499, 95% confidence interval: 1.055–1.944, *P*<0.05; D-loop: 0.643, 0.509–0.775 vs 0.925, 0.747–1.103, *P*<0.01; Figures 2d and e). Also, more patients in the ASPH-high group obtained somatic mutation in D-loop region compared with patients in the ASPH-low group (47.0%, 16/34 vs 18.9%, 7/30, *P*<0.05; Figure 2f). As such, the results suggested that ASPH expression level was associated with mtDNA contents and mutagenesis in HCC tissues.

### ASPH overexpression disrupts mtDNA stability in HCC cell lines

**Figure 3:**
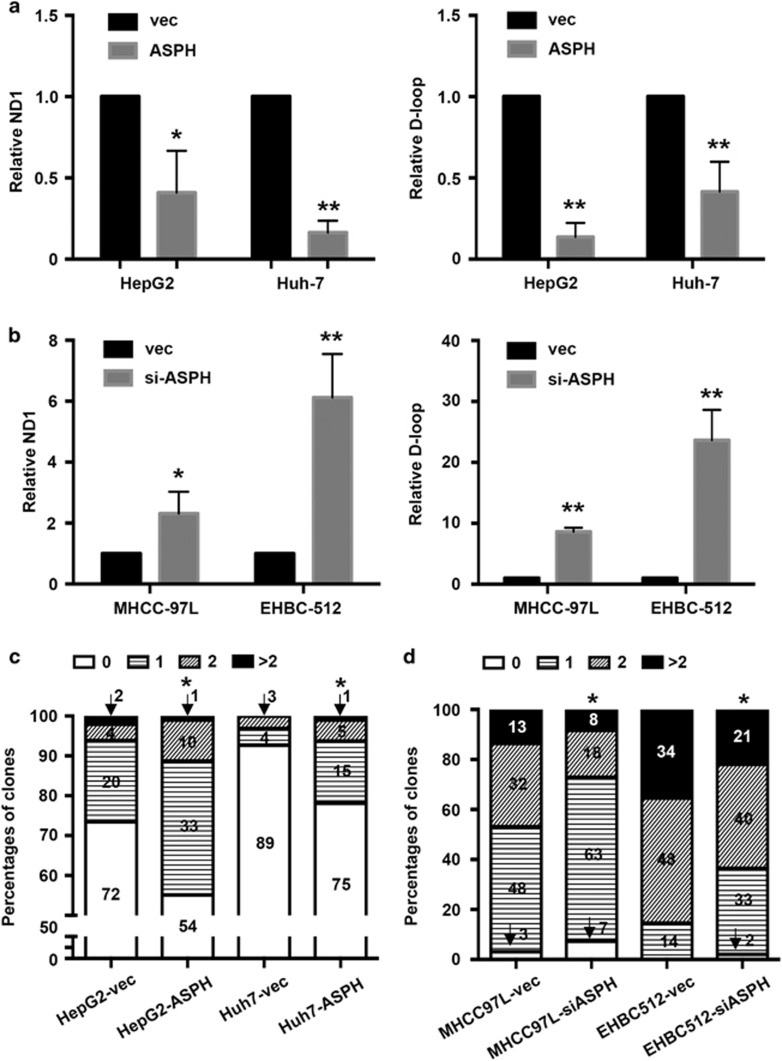
ASPH expression level affects mtDNA stability in HCC cell lines. (**a**) Alteration of relative ND-1 (left) and D-loop (right) copy number in the mtDNA of HepG2 and Huh-7 cells overexpressed with ASPH in comparison to the control cells. (**b**) Alteration of relative ND-1 (left) and D-loop (right) copy number in the mtDNA of MHCC-97 L and EHBC-512 cells with silenced ASPH in comparison to the control cells. For **a** and **b**, mtDNA copy number was assayed by real-time PCR and normalized to the amounts of β-actin DNA. (**c**) Percentages of sub-clones of HepG2 and Huh-7 cells that were overexpressed with ASPH or control vectors based on the amount of single-nucleotide variation in the D-loop region. (**d**) Percentages of sub-clones of MHCC-97 L and EHBC-512 cells that were overexpressed with ASPH siRNA or control vectors based on the amount of single-nucleotide variation in the D-loop region. For **c** and **d**, the D-loop mutation was identified by Sanger sequencing and the number of sub-clones in each group was marked in the corresponding column. All data are shown as mean±s.d. from at least three independent experiments. *P<0.05, **P<0.01 vs vector group. vec, ASPH and siASPH indicated cell lines overexpressed with vector, ASPH and ASPH siRNA, respectively.

To study the effect of ASPH expression level on mtDNA stability in HCC cells, ASPH expression level in HCC cell lines HepG2, Huh-7, MHCC-97 L and EHBC-512 was manipulated. ASPH was either overexpressed (HepG2-ASPH and Huh-7-ASPH) or silenced (MHCC-siASPH and EHBC-siASPH), followed by an evaluation on changes of copy number of D-loop and ND-1. As shown in Figure 3a, the ND-1 contents in HepG2-ASPH and Huh-7-ASPH decreased to 41.0±25.7% (*P*<0.05) and 16.4±7.3% (*P*<0.01), respectively, compared with the respective control cells; in contrast, the ND-1 contents in MHCC-siASPH and EHBC-siASPH increased to 231.3±71.8% (*P*<0.05) and 612.3±142.4% (*P*<0.01), respectively. D-loop copy numbers were also downregulated in HepG2-ASPH (13.6±8.6% in relative to HepG2-vec, *P*<0.01) and Huh-7-ASPH (41.6±18.4% in relative to Huh-7-vec, *P*<0.01), while upregulated in MHCC-siASPH (861.3±67.3% in relative to MHCC-vec, *P*<0.01) and EHBC-siASPH (2365.0±496.5% in relative to EHBC-vec, *P*<0.01; Figure 3b). Therefore, ASPH expression level affected mtDNA copy number.

In view of the heterogeneity of tumor cells within the cell lines, the D-loop regions of 96–98 monoclonal mtDNA for each cell type were then sequenced. As shown in Figures 3c, 73.5% (72/98), 20.4% (20/98), 4.1% (4/98) and 2.0% (2/98) of HepG2-vec cells had 0, 1, 2 and >2 mutations detected within the D-loop region, respectively. Nevertheless, the corresponding proportion of mutations was 55.1% (54/98), 33.7% (33/98), 10.2% (10/98) and 1.0% (1/98) in the sequenced HepG2-ASPH clones, suggesting an increased rate of mutagenesis in D-loop regions (*P*<0.05).

Similarly, the mutagenesis in D-loop region of Huh-7-ASPH cells was enhanced when compared with Huh-7-vec cells (Figure 3c). In contrast, when ASPH was silenced in MHCC-siASPH and EHBC-siASPH, fewer mutations in D-loop region were observed compared with the controls (Figure 3d). Thus, ASPH expression level also affected the mutagenesis in D-loop region.

### ASPH overexpression causes mitochondrial malfunction in HCC cell lines

**Figure 4:**
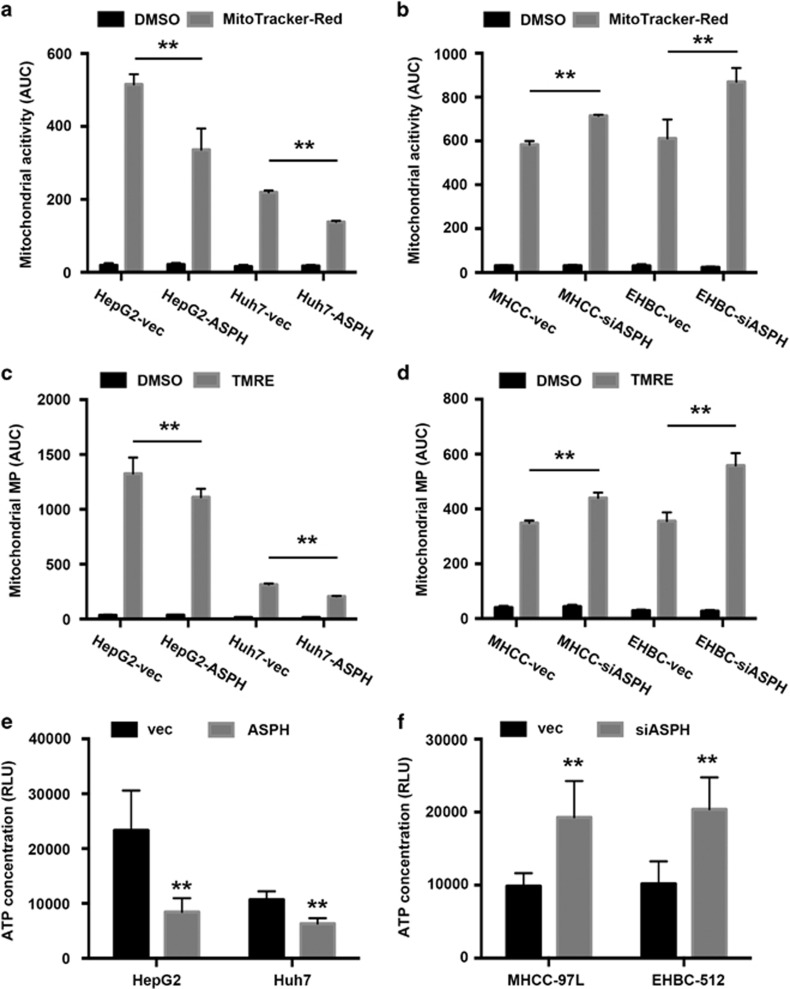
ASPH regulates mitochondrial function in HCC cell lines. (**a**, **b**) Mitochondrial activity assayed by MitoTracker Red staining and detected by flow cytometers in HepG2 and Huh-7 cells that was overexpressed with ASPH and control vectors, or MHCC-97 L and EHBC-512 cells that was overexpressed with ASPH siRNA and control vectors. The area under fluorescent count curve (AUC) was used for quantification. (**c**, **d**) Mitochondrial membrane potential assayed by tetramethylrhodamine ethyl ester (TMRE) staining and detected by flow cytometers in HepG2 and Huh-7 cells that was overexpressed with ASPH and control vectors, or MHCC-97 L and EHBC-512 cells that was overexpressed with ASPH siRNA and control vectors. The AUC was used for quantification. (**e**, **f**) Intracellular ATP concentration detected by a commercial bioluminescence assay in HepG2 and Huh-7 cells overexpressed with ASPH and control vectors, or MHCC-97 L and EHBC-512 cells overexpressed with ASPH siRNA and control vectors. Relative luminescence unit (RLU) was used for quantification. All data are shown as mean±s.d. from at least three independent experiments. **P<0.01, *P<0.05 vs vector group. vec, ASPH and siASPH indicated cell lines overexpressed with vector, ASPH and ASPH siRNA, respectively.

We next investigated whether disruption of mtDNA integrity caused by ASPH overexpression was associated with mitochondrial malfunction. We assayed and compared cellular mitochondrial activity among different HCC cells stained with MitoTracker dye by flow cytometers. Using the area under curve as a quantitative index, ASPH overexpression resulted in 35% and 37% reduced mitochondrial activities in HepG2 and Huh-7 cells compared with the matched control cells, respectively (HepG2-ASPH vs HepG2-vec: 336.1±58.1 vs 515.0±28.2, *P*<0.01; Huh-7-ASPH vs Huh-7-vec: 138.3±2.8 vs 219.3±4.6, *P*<0.01; Figure 4a; Supplementary Figure S3a). In contrast, silencing ASPH enhanced mitochondrial activities by 22% and 42% in MHCC-97 L and EHBC-512 cells compared with the control group, respectively (MHCC-siASPH vs MHCC-vec: 714.5±5.4 vs 582.8±16.9, *P*<0.01; EHBC-siASPH vs EHBC-vec: 869.7±63.5 vs 611.2±86.5, *P*<0.05; Figure 4b; Supplementary Figure S3b).

ASPH overexpression consistently and markedly reduced the area under curve of mitochondrial membrane potential in HepG2 cells (HepG2-ASPH vs HepG2-vec: 1111.3±76.2 vs 1326.7±146.1, *P*<0.01) and Huh-7 cells (Huh-7-ASPH vs Huh-7-vec: 206.3±5.1 vs 314.5±8.8, *P*<0.01; Figure 4c; Supplementary Figure S3c). Knocking down of ASPH promoted the potential in MHCC-97 L cells (MHCC-siASPH vs MHCC-vec: 440.0±19.5 vs 349.2±8.6, *P*<0.01) and EHBC-512 cells (EHBC-siASPH vs EHBC-vec: 558.5±44.9 vs 355.3±32.2, *P*<0.01; Figure 4d; Supplementary Figure S3d). Therefore, the mitochondrial membrane potential could be affected by ASPH expression level.

Consistent with aberrant mitochondrial activity and membrane potential, the intracellular ATP concentration in HepG2 and Huh-7 cells overexpressed with ASPH was lower compared with control cells, characterized by markedly less relative luminescence unit in the ATP detection assay (HepG2-ASPH vs HepG2-vec: 8438.6±2500.5 vs 23311.2±7268.3, *P*<0.01; Huh-7-ASPH vs Huh-7-vec: 6310.8±1006.5 vs 10674.6±1531.6, *P*<0.01; Figure 4e). Conversely, downregulation of ASPH promoted ATP generation in MHCC-97 L cells (MHCC-siASPH vs MHCC-vec: 19290.6±4963.2 vs 9868.8±1773.7, *P*<0.01) and EHBC-512 cells (EHBC-siASPH vs EHBC-vec: 20388.6±4367.2 vs 10184.2±3087.3, *P*<0.01; Figure 4f). Thus, ASPH affected intracellular ATP generation likely though regulating mitochondrial membrane potential.

### ASPH overexpression increases ROS

**Figure 5:**
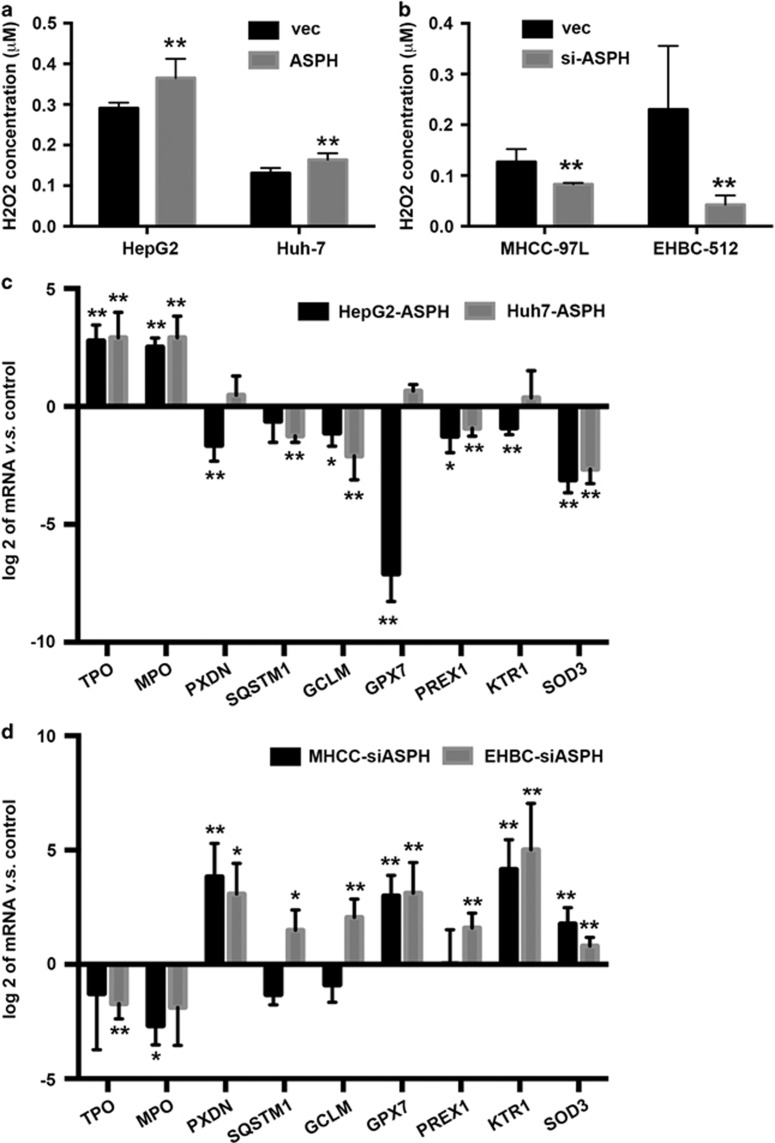
ASPH regulates ROS generation in HCC cell lines. (**a**, **b**) Intracellular ROS generation characterized and assayed by intracellular H2O2 concentration and detected by flow cytometers in HepG2 and Huh-7 cells overexpressed with ASPH and control vectors, or MHCC-97 L and EHBC-512 cells overexpressed with ASPH siRNA and control vectors. (**c**, **d**) Ratios of mRNA expression level of multiple genes associated with ROS generating and chelating detected by real-time PCR in HepG2 and Huh-7 cells overexpressed with ASPH, or MHCC-97 L and EHBC-512 cells with silenced ASPH to the control groups. All data are shown as mean±s.d. from at least three independent experiments. **P<0.01, *P<0.05 vs vector group. vec, ASPH and siASPH indicated cell lines overexpressed with vector, ASPH and ASPH siRNA, respectively.

In addition to energy metabolism, ROS homeostasis is another important biological function for mitochondria. The exogenously enforced expression of ASPH in HepG2 and Huh-7 cells caused an increase of ROS that was characterized by upregulation of intracellular H2O2 concentration as compared with the controls (HepG2-ASPH vs HepG2-vec: 0.365±0.047 vs 0.291±0.014, *P*<0.01; Huh-7-ASPH vs Huh-7-vec: 0.164±0.016 vs 0.131±0.012, *P*<0.01; Figure 5a). Conversely, loss of ASPH via gene silencing in MHCC-97 L and EHBC-512 cells resulted in a reduced ROS level in comparison with MHCC-vec cells (0.414±0.014 vs 0.634±0.127, *P*<0.01) and EHBC-vec cells (0.212±0.092 vs 1.151±0.626, *P*<0.01; Figure 5b). Thus, the intracellular ROS level could be also influenced by ASPH expression level in HCC cell lines.

To investigate the influence of ASPH expression on ROS generation and chelation signaling, mRNA expression of multiple genes was screened in relation to ROS homeostasis using a PCR array (Supplementary Table S4) and then validated the results using real-time PCR. These genes included thyroid peroxidase (*TPO*), myeloperoxidase (*MPO*), peroxidasin homolog (*PXDN*), sequestosome 1 (*SQSTM1*), glutamate–cysteine ligase modifier subunit (*GCLM*), phosphatidylinositol-3,4,5-trisphosphate-dependent Rac exchange factor 1 (*PREX1*), keratin 1 (*KRT1*) and superoxide dismutase 3 (*SOD3*). We found that ASPH overexpression promoted biosynthesis of ROS generation enzymes in HepG2 and Huh-7 cells, such as TPO and MPO. Similarly, we observed a reduced expression of enzymes capable of cleaning up ROS, such as GCLM, PREX1 and SOD3 (Figure 5c). In support of these findings, downregulation of ASPH in MHCC-97 L and EHBC-512 displayed an opposite effect (Figure 5d). Therefore, ASPH overexpression induced the alteration of gene expression patterns that promoted ROS generation while inhibited ROS elimination.

### ASPH competes with transcription factor A in interacting with histone H2A member X

To identify ASPH-mediated downstream signals regulating mtDNA stability, we analyzed the compositions of protein complex immunoprecipitated by ASPH antibodies, in which a mitochondrial localized protein, histone H2A member X (H2AX), emerged as a candidate.

**Figure 6:**
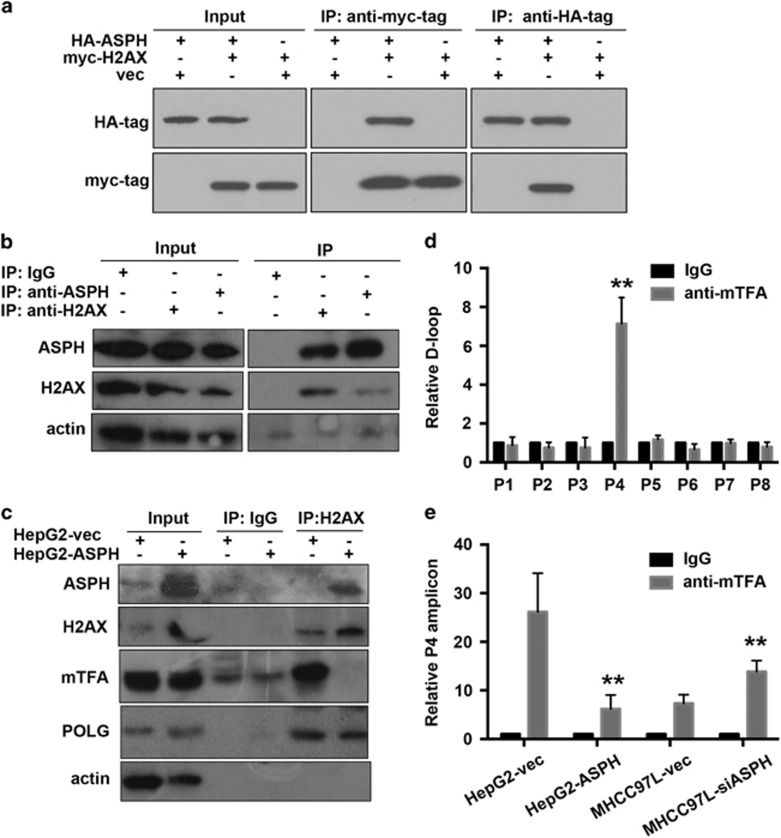
ASPH competes with mtTFA in interacting with H2AX in HCC cell lines. (**a**) Interaction between exogenously expressed HA-tagged ASPH and myc-tagged H2AX in 293 cells assayed by immunoprecipitation using HA antibody or myc antibody. (**b**) Interaction of endogenous ASPH and H2AX in MHCC-97 L cells assayed by immunoprecipitation using ASPH antibody or H2AX antibody. (**c**) Influence of ASPH overexpression on mTFA–H2AX interaction. The protein complex that was immunoprecipitated by H2AX antibody in HepG2 cells overexpressed with ASPH and control vectors was further analyzed by immunoblot using ASPH, POLG and mTFA antibodies. (**d**) Identification of mTFA-binding mtDNA motif in the D-loop region through a chromosome immunoprecipitation (ChIP)-PCR assay. The protein–DNA complex was immunoprecipitated by mTFA antibody in MHCC-97 L cells. The putative bound mtDNA motif was further amplified using eight primer pairs that covered the D-loop region and were designated from P1 to P8. The amplicon of primer set 4 (P4) showed enrichment in the protein–DNA motif. **P<0.01 vs IgG group. (**e**) Influence of ASPH overexpression on mTFA-binding probability to D-loop region through a ChIP-PCR assay. The amounts of D-loop region characterized by P4 amplicon in the mTFA–DNA complex were analyzed in HepG2 cells overexpressed with ASPH or MHCC-97 L cells with silenced ASPH in comparison to the control group overexpressed with vectors. All data are shown as mean±s.d. from at least three independent experiments. **P<0.01 vs vector group.

Co-immunoprecipitation assay consistently showed that hemagglutinin (HA)-tagged ASPH and myc-tagged H2AX were in a common protein complex (Figure 6a). Exogenous ASPH–H2AX interaction was also confirmed in mitochondrial fraction by co-immunoprecipitation assay (Supplementary Figure S4a). In addition, we detected endogenous H2AX or ASPH presence in immunoprecipitant by ASPH or H2AX antibodies in total cell lysates (Figure 6b) and mitochondrial fraction (Supplementary Figures S4b and c). We then examined whether the interaction between H2AX and mitochondrial transcription factor A (mtTFA) or DNA polymerase gamma (POLG) could be affected by ASPH–H2AX interaction. As shown in Figure 6c, ASPH could interact with H2AX in HepG2-ASPH cells, whereas the interaction between mtTFA and H2AX was greatly diminished compared with that in HepG2-vec cells. Of note, ASPH overexpression did not affect POLG–H2AX interaction and failed to influence mtTFA expression level. Thus, we propose that ASPH competes with mtTFA in interacting with H2AX in HCC cell lines.

To clarify the outcome of weakened H2AX–mtTFA interaction caused by ASPH overexpression, we tested the binding efficiency of mtTFA to D-loop region. The mtTFA-binding area was screened by amplifying mtTFA-binding fragment using tilling primers covering whole D-loop regions. As shown in Figure 6d, the amplicon amplified by P4 primer set showed a significant enrichment in mtDNA fragment pulled down by mtTFA antibodies when compared with IgG isotype. This amplicon was used as reporter of mtTFA-binding efficiency to mtDNA.

We then examined the content of D-loop region amplified by P4 primer set in HCC cells with varied ASPH expression level and found that ASPH overexpression greatly impaired mtTFA binding to D-loop region in HepG2 cells (HepG2-ASPH vs HepG2-vec: 6.2±2.9 vs 26.1±8.0, *P*<0.01), while ASPH silencing facilitated mtTFA binding in MHCC-97 L cells (MHCC-siASPH vs MHCC-vec: 13.9±2.2 vs 7.3±1.8, *P*<0.01; Figure 6e). Collectively, ASPH could influence mtTFA-binding probability to mtDNA in HCC cell lines.

## Discussion

In this study, we describe a previously unidentified function of ASPH in disrupting mtDNA stability and mitochondrial function. The data demonstrate that ASPH localizes on mitochondria in HCC cells and HCC tissues and interacts with H2AX. Overexpression of ASPH disrupts the mtDNA integrity through H2AX–mtTFA signal, thereby affecting mitochondrial functions in HCC. The reduction of mtDNA has been observed in some human neoplasms, such as renal carcinoma, gastric cancer, breast cancer and HCC.^28, 29, 30, 31^ We here demonstrated that the changes in mtDNA copy number were associated with aggressive pathological features of HCC, including large tumor size, microvascular invasion, absence of tumor capsule and advanced disease stage. These data suggested an important role of mtDNA instability in disease development.

The underlying mechanism of mtDNA aberrance in HCC remains uncharacterized, though the involvement of mtDNA instability in carcinogenesis has been well described.^12, 13, 14^ On the other hand, ASPH has been recognized as an important molecular marker associated with tumor invasion and disease outcome in some types of cancers, including HCC,^23, 24, 25, 32, 33, 34^ but the related mechanism and downstream signaling are largely unknown. In the current study, we noted mitochondrial localization of ASPH in HCC cells using an antibody specific for the catalytic domain of ASPH. The data further highlighted a role of mitochondrial ASPH in regulating mtDNA copy numbers and somatic mtDNA mutation. Both of these features are hallmarks of mtDNA instability in HCC.^12, 13, 19, 28^ ASPH overexpression in HepG2 and Huh-7 cells significantly increased mutation frequency in the D-loop region and decreased copy numbers of D-loop and ND-1 region, the most frequently altered mtDNA region during tumorigenesis. These *in vitro* results were consistent with our correlation study results between ASPH expression level and mtDNA integrity in HCC tissues, which demonstrated that HCC tissues with high ASPH expression showed less mtDNA copy numbers while more mutations compared with tissue that had low ASPH expression.

The mtDNA instability induced by ASPH overexpression might contribute to mitochondrial dysfunction in HCC cells. We found that ASPH overexpression decreased mitochondrial membrane potential and mitochondrial activity, and resulted in a decreased ATP generation. Simultaneously, the intracellular ROS levels within those cells were greatly enhanced, mainly due to irregular activity of ROS generating and chelating pathway. These consequences might be a direct result of the effect of ASPH on mtDNA integrity, as reduction in the copy number of mtDNA and somatic mutations in D-loop region were accompanied with a reduction of mitochondrial respiratory enzymes, thereby repressing the function of mitochondria and influencing ROS production.^13^ Of note, it has been reported that increasing ROS could promote ASPH expression,^35^ which inevitably worsen mtDNA instability.

We newly identified the interplay between ASPH and mitochondrial H2AX by a high-throughput proteomic strategy of mass spectrometry. H2AX has been identified as a protein that localizes to the mitochondria and serves as a shuttle protein transporter involved in mitochondrial protein transport.^36^ This interaction might facilitate the mitochondrial localization of ASPH, which needs to be further investigated. Moreover, the increased occupation of H2AX by ASPH binding might affect other cargo proteins loading to this transporter. In this study, we detected a decreased mtTFA, a previously identified mitochondrial protein shuttled by H2AX,^36^ in the immunoprecipitated H2AX protein complex of HCC cells overexpressing ASPH. The mtTFA has been widely known as one of the most important members for mtDNA stability involved in mtDNA replication, transcription and repair.^37, 38, 39^ The weakened H2AX–mtTFA interaction caused by ASPH overexpression might result in two outcomes. First, the ability of mtTFA to translocate to the mitochondria, where mtDNA replication mainly occurs, might be greatly abolished via H2AX–ASPH interactions. Another consequence is that mitochondrial mtTFA might not be capable of forming a proper protein complex to initial mtDNA replication, for which mtTFA–H2AX interaction itself is also necessary.^36^ The mtDNA immunoprecipitation data demonstrated that mtTFA binding to the D-loop region is greatly reduced by ASPH overexpression in HCC cells. Therefore, H2AX and mtTFA might be the central molecules mediating the function of mitochondrial ASPH in mtDNA integrity.

The current study had several limitations. As a hydroxylase, whether the enzymatic activity of ASPH is required for its role in regulating mtDNA integrity remains to be investigated in the future study. In addition, the essential role of mtTFA in mtDNA replication has been well established, whereas its contribution to mtDNA mutagenesis requires more investigation.

In conclusion, we reported the mitochondrial localization of ASPH and its role in mtDNA instability and mitochondrial dysfunction in HCC. In the proposed model (Supplementary Figure S5), ASPH localizes to the mitochondria where it interacts with H2AX. This interaction interferes with H2AX–mtTFA binding in a competitive manner. The disruption of H2AX–mtTFA may prevent mtTFA from binding to mtDNA. As an overall outcome, overexpression of ASPH causes a reduced mtDNA copy number and an increased mtDNA mutation. Findings from the current study expand the mechanistic understanding of mitochondrial malfunction in HCC and suggest ASPH as an intervention target for maintaining mtDNA stability.

## Materials and methods

### Patients and tissue samples

The study was approved by the Institutional Ethics Committee of the Eastern Hepatobiliary Surgery Hospital, and informed consent was obtained from each patient to use their data and surgically resected specimens in this study.

Surgical specimens, including tumor and paired non-tumor tissues, from 140 patients who underwent liver resection for histologically proven HCC at the Eastern Hepatobiliary Surgery Hospital between March 2011 and June 2011 were collected and used in this study.

As previously reported,^25^ patients were grouped according to the level of ASPH expression. Specifically, the ratio of ASPH mRNA in the tumor tissues as compared with the matched non-tumor control tissues was tested by real-time PCR. Among 140 HCC patients, 34 patients had ASPH high expression (ratio>2), while 37 patients had ASPH low expression in tumor tissues (ratio<0.5). High or low expression of ASPH was also confirmed by immunoblotting (Supplementary Figure S6). These 71 patients were further used to study the correlation between mtDNA integrity, ASPH expression level and the clinicopathological features. The clinicopathological data of these patients are listed in Supplementary Table S2.

### Cell cultures

Human embryonic kidney 293 cell and human liver cancer cell lines included HepG2, Huh-7, MHCC-97 L and EHBC-512 were maintained as previously described.^40^ Additional information on cell culture is detailed in the Supplementary Information.

### Constructs

The cDNA encoding the full open reading frame sequence of ASPH and H2AX was purchased from Origene (Rockville, MD, USA). The siRNA sequences for silencing ASPH was 5′-GCGCAGTGTGAGGATGAT-3′. We also sub-cloned expression constructs of HA-tagged ASPH or myc-tagged H2AX by adding a HA or myc tags at the N terminus of ASPH or H2AX, respectively. The lentivirus of wild-type ASPH and the siRNAi against human ASPH were purchased from Sunbio (Beijing, China).

### Antibodies

A polyclonal antibody against ASPH was raised in our institution using the synthetic peptide antigen of 12 amino-acid residues around the Fe^2+^-binding domain of ASPH. The antibodies against mitochondrial biomarkers heat shock protein 60 (ab46798) and voltage-dependent anion channel (ab154856), endoplasmic reticulum biomarkers calnexin (ab195198), green fluorescent protein (ab6556), H2AX (ab11175), POLG (ab207558) and mtTFA (ab176558) were purchased from Abcam (Cambridge, UK). The mouse monoclonal anti-myc (9E10) and HA antibodies (12CA5) were purchased from Sigma-Aldrich (St Louis, MO, USA) and Roche (Basel, Switzerland), respectively. Additional information on secondary antibodies is detailed in the Supplementary Information.

### Subcellular fraction preparation

The mitochondrial fraction of cultured cells was isolated using Mitochondria Isolation Kit (Thermo) (Waltham, MA, USA) according to the specifications provided by the manufacturer. The cytosol and mitochondrial fractions were further lysed with 2% 3-[(3-cholamidopropyl)dimethylammonio]propanesulfonate (CHAPS) in Tris-buffered saline, boiled with SDS–polyacrylamide gel electrophoresis sample buffer and applied for gel electrophoresis.

### Immunoblot and immunofluorescence staining

Immunostaining and immunoblot were performed as previously described.^40^ Fluorescent images were taken using a confocal microscope of FV1000 (Olympus, Tokyo, Japan). Hoechst 33258 (Molecular Probes, Waltham, MA, USA) was used for nuclear staining.

### PCR array and real-time PCR

The Human Oxidative Stress RT2 Profiler PCR Array Kit (Qiagen, Hilden, Germany) was used to analyze the ROS metabolism-related gene expression in HepG2 cells overexpressing ASPH, MHCC-97 L cells with ASPH silenced and their respective controls. RNA extraction and cDNA preparation were performed as previously described.^40^ The PCR array was performed according to the protocol recommended by the manufacturer. Differentially expressed genes were confirmed by real-time PCR. The primers are listed in Supplementary Table S3. β-actin served as an internal control. Additional information on PCR procedures is detailed in Supplementary Information.

### mtDNA copy number and somatic mutation detection

The amounts of ND-1 and D-loop fragment to reflect the changes of copy number of mtDNA were assayed as previously described.^12, 13^ The copy numbers of ND-1 or D-loop in the mtDNA of HCC samples were defined as the ratio between tumor and matched non-tumor control. The copy numbers of ND-1 or D-loop in the mtDNA of HCC cells were quantified after normalization to internal control. The primers are listed in Supplementary Table S3. β-actin served as an internal control. Additional information on PCR procedures is detailed in the Supplementary Information.

To detect mutations in the D-loop region of mtDNA, we used mtDNA extracted from paired HCC tissues or multiple clones of HCC cell lines cultured from single tumor cells to reflect the intrinsic heterogeneity of mtDNA. The D-loop region was amplified as previously described,^41^ using primers listed in Supplementary Table S3. Additional information on PCR procedures is detailed in the Supplementary Information.

### ROS and ATP generation measurement

The intracellular ROS generation in HCC cell lines was measured through Amplex Red Hydrogen Peroxide/Peroxidase Assay Kit (Life Invitrogen, Waltham, MA, USA, A22188). The intracellular ATP generation in HCC cell lines was measured using ATP Determination Kit (Biovision, Milpitas, CA, USA). All assays were performed according to the manufacturer’s instructions. Additional information on assay procedures is detailed in the Supplementary Information.

### Mitochondrial membrane potential and mitochondrial activity measurement

Mitochondrial activity and membrane potential of HCC cell lines were, respectively, measured using MitoTracker Red CMXRos (MTR, Life Invitrogen) or tetramethylrhodamine ethyl ester (Life Invitrogen) following the manufacturer’s instructions.

### Mass spectrometry and immunoprecipitation

The FLAG-ASPH plasmids were transfected into 293 cells. Crude cell lysate was prepared 72 h after transfection. The protein complex interacting with FLAG-ASPH was obtained using the FLAG HA Tandem Affinity Purification Kit (Sigma-Aldrich), according to the manufacturer’s instructions. Mass spectrometry analysis was performed on flag-tagged immunoprecipitant. The data were analyzed by Research Center for Proteome Analysis, Shanghai, China. Co-immunoprecipitation was performed using the cell lysates or the mitochondrial fraction from 293 cells co-transfected with HA-ASPH and myc-H2AX. The analysis of endogenous interaction among ASPH, H2AX and mtTFA was performed in HCC cell lines using their respective antibodies.

### mtDNA immunoprecipitation and PCR

The procedures of mtDNA immunoprecipitation were slightly modified from conventional chromatin immunoprecipitation. A unit of 1 mg of pre-cleared mitochondrial protein was mixed with 20 μg anti-mtTFA antibody and AminoLink Plus Resin (Thermo Fisher Scientific, Waltham, MA, USA) by end-over-end inversion overnight at 4 °C. Antibodies of IgG isotype served as a control. The immunoprecipitant was centrifuged, washed and crosslinked. The bound DNA was extracted using the QIAamp DNA mini kit (Qiagen) according to the manufacturer’s instructions. We designed eight pairs of primers across the whole mtDNA D-loop region for PCR amplification. The primers are listed in Supplementary Table S3.

### Statistical analysis

All statistical analysis was performed using SPSS (version 18.0, Chicago, IL, USA). Continuous variables were expressed as median (range) and compared using the nonparametric Mann–Whitney *U*-test, Kruskal–Wallis test and two-independent samples *t*-test or paired *t*-test. Categorical variables were reported as the number of cases and the prevalence, and the differences between the groups were compared using the *χ*^2^-test with Yates correction or Fisher exact test as appropriate. A *P*<0.05 was considered as statistically significant.
