# Supplementary material for: Aspartate β-hydroxylase disrupts mitochondrial DNA stability and function in hepatocellular carcinoma
Source: Oncogenesis. 2017 Jul 17;6(7):e362–. doi: 10.1038/oncsis.2017.64 (PMC5541716; doi:10.1038/oncsis.2017.64)
Supplement: Supplementary Information [file oncsis201764x1.doc]

**Supplementary Information**

**Supplementary Materials and Methods**

***Cell cultures***

Human liver cancer cell lines, HepG2, Huh-7, MHCC-97L and EHBC-512, were maintained and used in this study. Briefly, cells were cultured in Dulbecco’s modified Eagle medium (Invitrogen) supplemented with 10% fetal bovine serum (Thermo Fisher Scientific) and antibiotics (penicillin and streptomycin, 50 units/ml each, Invitrogen) in 5% CO_2_ atmosphere at 37°C.

***Antibodies***

Horseradish peroxidase–conjugated secondary antibodies, polyclonal goat anti-rabbit IgG and goat anti-mouse IgG were used at 1:5000 dilutions (GE Healthcare). Alexa Fluor 546 goat anti rabbit IgG, Alexa Fluor 594 goat anti mouse IgG, Alexa Fluor 488 goat anti rabbit IgG, Alexa Fluor 633 goat anti mouse IgG were purchased from Life Technologies (Invitrogen).

***PCR array and Real-time PCR***

The PCR-array was performed according to the protocol recommended by the manufacturer (Qiagen). The differential genes revealed by the PCR-array were further confirmed by real-time PCR that was performed with SYBR Premix Ex Taq Kit (TaKaRa) and analyzed on ABI PRISM 7000 Sequence Detection System (Applied Biosystems).

***mtDNA copy number and somatic mutation detection***

As for detecting mtDNA copy number, the genomic DNA was extracted using genomic DNA isolation Kit (Biovision) according to the protocol provided by the manufacturer. PCR was performed with SYBR Premix Ex Taq Kit (TaKaRa,) and analyzed on ABI PRISM 7000 Sequence Detection System (Applied Biosystems). The primers are listed in Supplementary information, Table S3. β-actin served as an internal control.

The primer used for D-loop region amplification in this assay is shown in the Supplementary information, Table S3. PCR was carried out in 25 μl total reaction volumes, each containing 1μl of template DNA, 0.2 pM of each primer, 2.5 μl of 10×PCR buffer, 1.5 mM MgCl2, 200mM dNTPs, and 1unit of Tag DNA polymerase (Fermentas). The reaction procedures were as followed: initial denaturation under 94°C for 5 minutes, followed by 30 cycles consisting of 1minute denaturation at 94°C, 1 minute annealing at 60°C, 1 minute of extension at 72°C and a final 5 minutes extension at 72°C. The PCR amplification products were subjected to purification in a 1% agarose gel for further Sanger sequencing.

***ROS and ATP generation measurement***

The amount of ROS, characterized by H2O2 concentration, was eventually evaluated by the absorbance with excitation at 540 nm and emission detection at 590 nm that was read out in fluorescence microplate reader and further fitted to the standard curve. All experiments were performed as duplicate for at least 6 times.

The intracellular ATP generation in HCC cell lines was measured through ATP Determination Kit (Biovision) according to the protocol provided by the manufacturer. Briefly, a volume of 10 μl crude cell lysate from 1×10^6^ HCC cells were added to 100 μl standard reaction system each containing 0.5 μl 20X reaction buffer (Component E­), 0.1 μl 0.1 M DTT, 0.5 μl of 10 mM D-luciferin, 2.5 μl of firefly luciferase from 5 mg/mL stock solution. The ATP generation was evaluated by the fluorescence intensity that was read out in fluorescence microplate reader and further fitted to the standard curve. All experiments were performed as duplicate for at least 6 times.

**Supplementary Figures and Legends**

**Supplementary information, Figure S1. The endogenous expression of ASPH in different HCC cell lines.**

The endogenous mRNA expression of ASPH in HepG2, Huh-7, MHCC-97L and EHBC-512 cells was evaluated by qPCR.

**
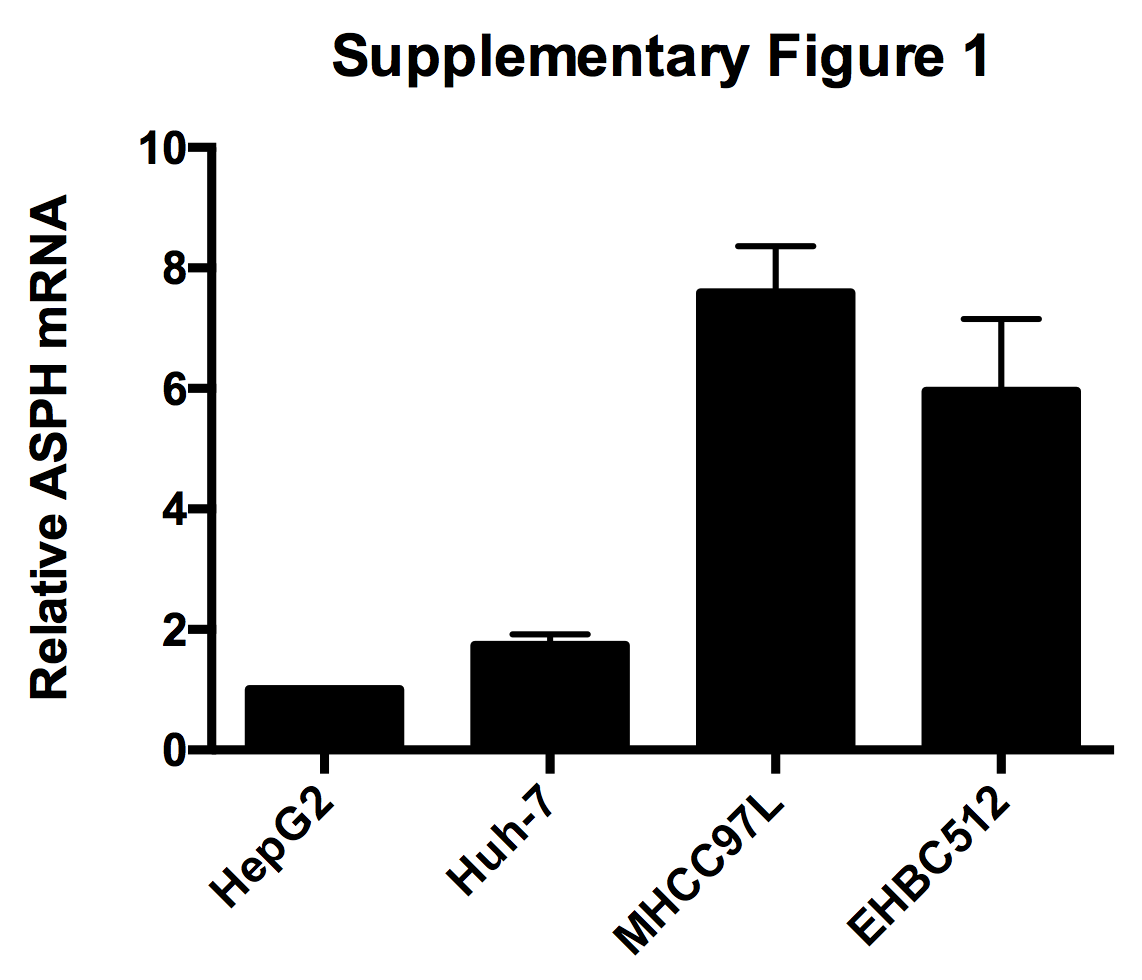
**

**Supplementary information, Figure S2. ASPH localizes on mitochondria in HCC tissues.** The cytoplasmic and mitochondrial fraction of HCC tissues were isolated using Tissue Mitochondria Isolation Kit (C3606, Beyotime) according to the specifications provided by the manufacturer and immunostained with anti-ASPH, anti-VDAC and anti-calnexin antibodies. The results are representative of at least three experiments. *mito*: fraction of mitochondrial protein; *cyto*: fraction of cytoplasmic proteins. VDAC is biomarker of mitochondrial organelles. Calnexin is a biomarker of endoplasmic reticulum.

**
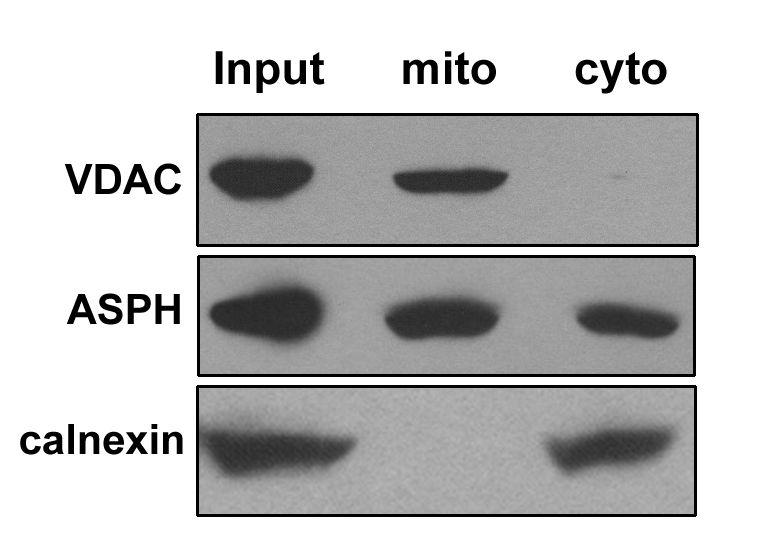
**

**Supplementary information, Figure S3.** ASPH regulates mitochondrial function in HCC cell lines. (**a** and **b**) Mitochondrial activity assayed by MitoTracker-Red (MTR) staining and detected by flow cytometers in HepG2 and Huh-7 cells that was over-expressed with ASPH and control vectors or MHCC-97L and EHBC-512 cells that was over-expressed with ASPH siRNA and control vectors. (**c** and **d**) Mitochondrial membrane potential assayed by TMRE staining and detected by flow cytometers in HepG2 and Huh-7 cells that was over-expressed with ASPH and control vectors or MHCC-97L and EHBC-512 cells that was over-expressed with ASPH siRNA and control vectors. The results are representative of at least three experiments. As abbreviations, *vec*, *ASPH* and *si-ASPH* indicated cell lines over-expressed with vector, ASPH and ASPH siRNA, respectively.

**
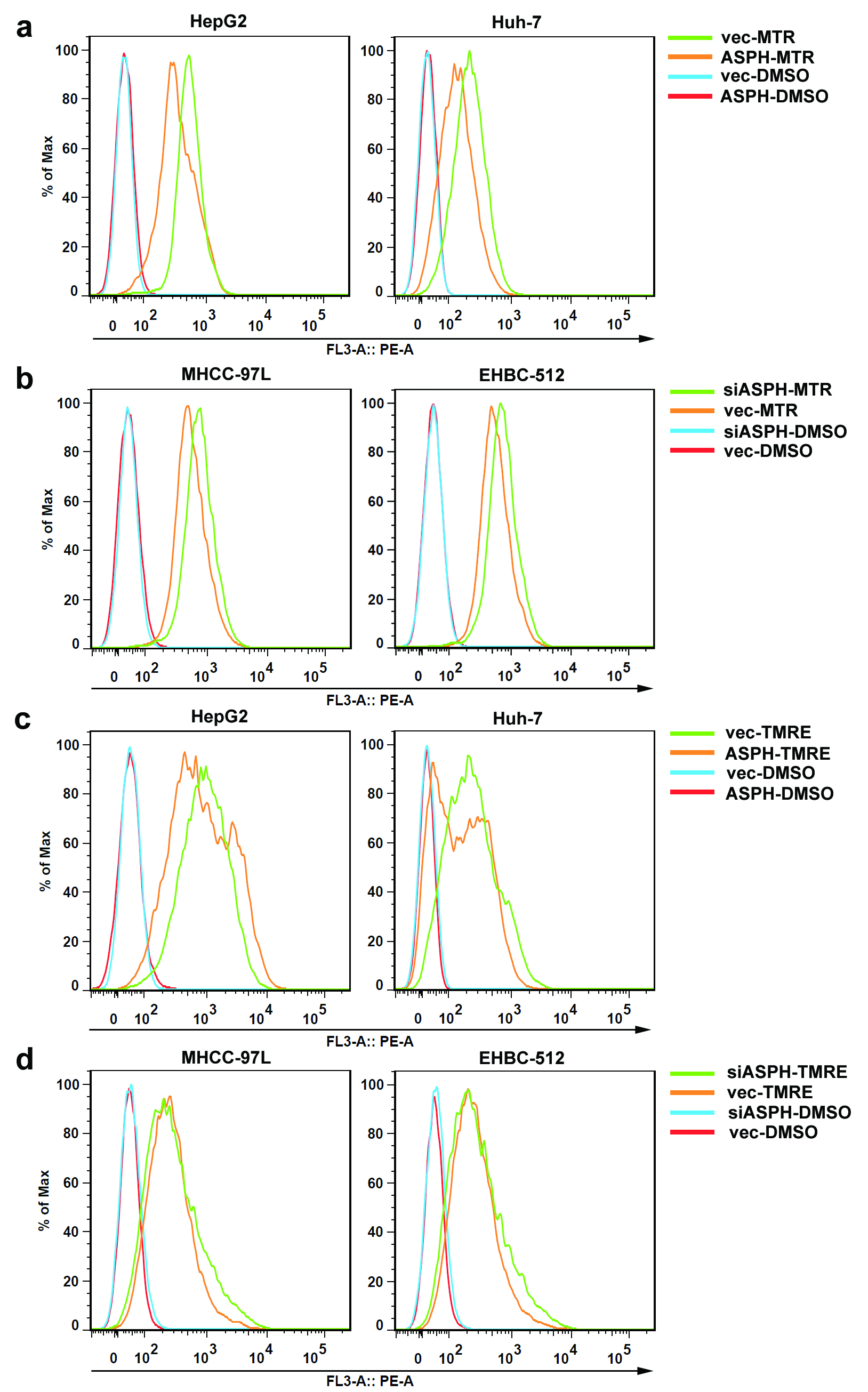
**

**Supplementary information, Figure S4. ASPH interacts with H2AX in mitochondrial fraction.** (**a**) HA-tagged ASPH and myc-tagged H2AX were overexpressed in 293 cells. The mitochondrial fraction of cultured cells was isolated using Mitochondria Isolation Kit (Thermo) according to the specifications provided by the manufacturer. Co-immunoprecipitation was performed using the mitochondrial fraction from 293 cells co-transfected with HA-ASPH and myc-H2AX. (**b**) The mitochondrial fraction of MHCC-97L cells was isolated using Mitochondria Isolation Kit (Thermo) according to the specifications provided by the manufacturer. The mitochondrial and cytoplasmic fractions of MHCC-97L cells were analyzed by immunoblot using VDAC and calnexin antibodies. (**c**) Interaction of endogenous ASPH and H2AX in mitochondrial fraction of MHCC-97L cells was assayed by immunoprecipitation using ASPH antibody or H2AX antibody. The results are representative of at least three experiments. For (b), *mito*: fraction of mitochondrial protein; *cyto*: fraction of cytoplasmic proteins. VDAC is a biomarker of mitochondrial organelles. Calnexin is a biomarker of endoplasmic reticulum.

**
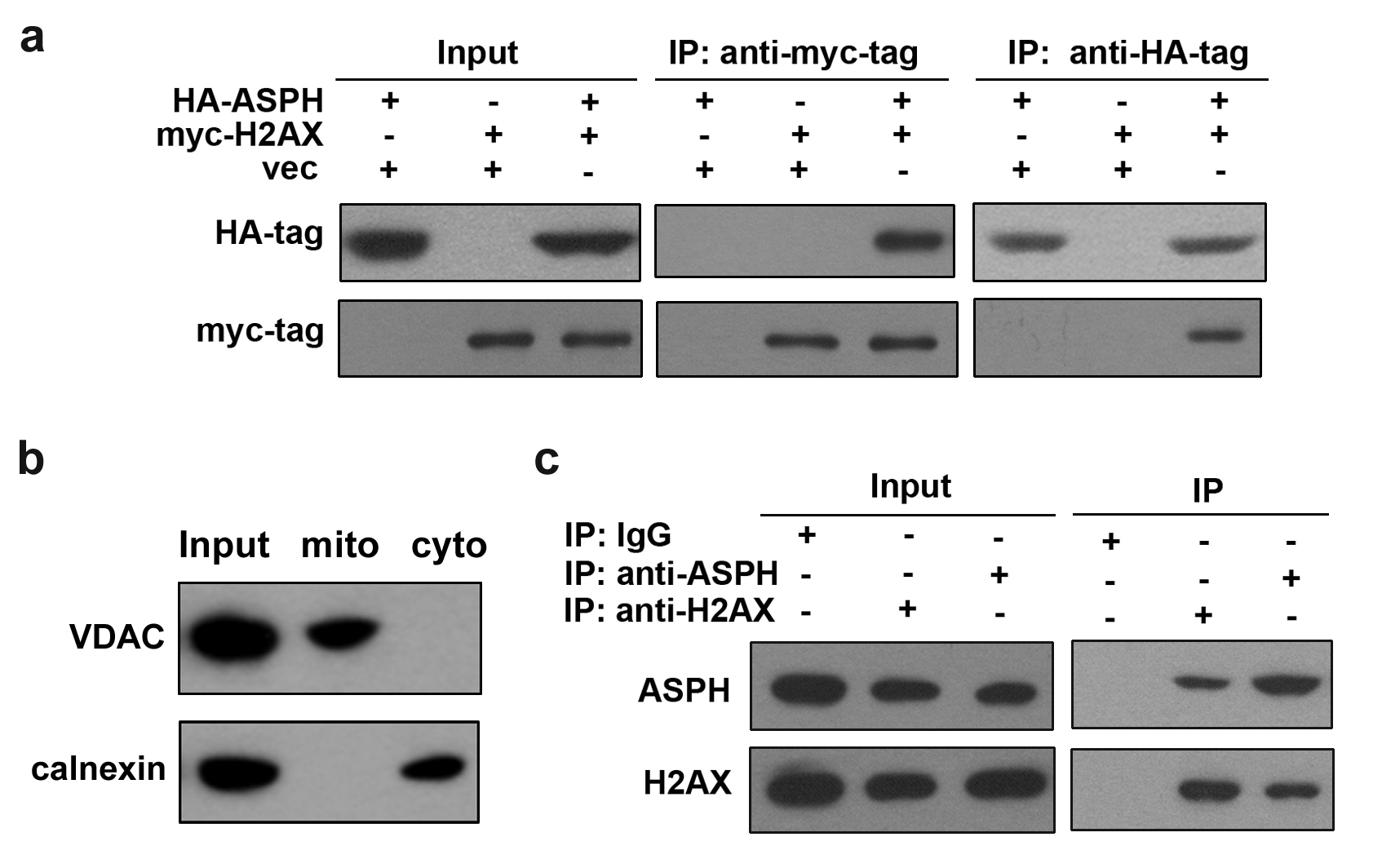
**

**Supplementary information, Figure S5. Schematic representation of the proposed ASPH-H2AX-mTFA signaling in regulating mtDNA integrity.**

In normal cells, mitochondrial H2AX interacts with mTFA, which facilitates mTFA to traffic to the mitochondrial and mediates mtDNA replication. However, in HCC cells, ASPH is over-expressed in mitochondria, which competitively interacts with H2AX. As a result, the interplay between H2AX and mtTFA is weakened, which prevents mtTFA properly bind to mtDNA for its function. The mtDNA integrity is thereby disrupted and eventually results in aberrant mitochondrial functions, such as abnormal ROS production.

**
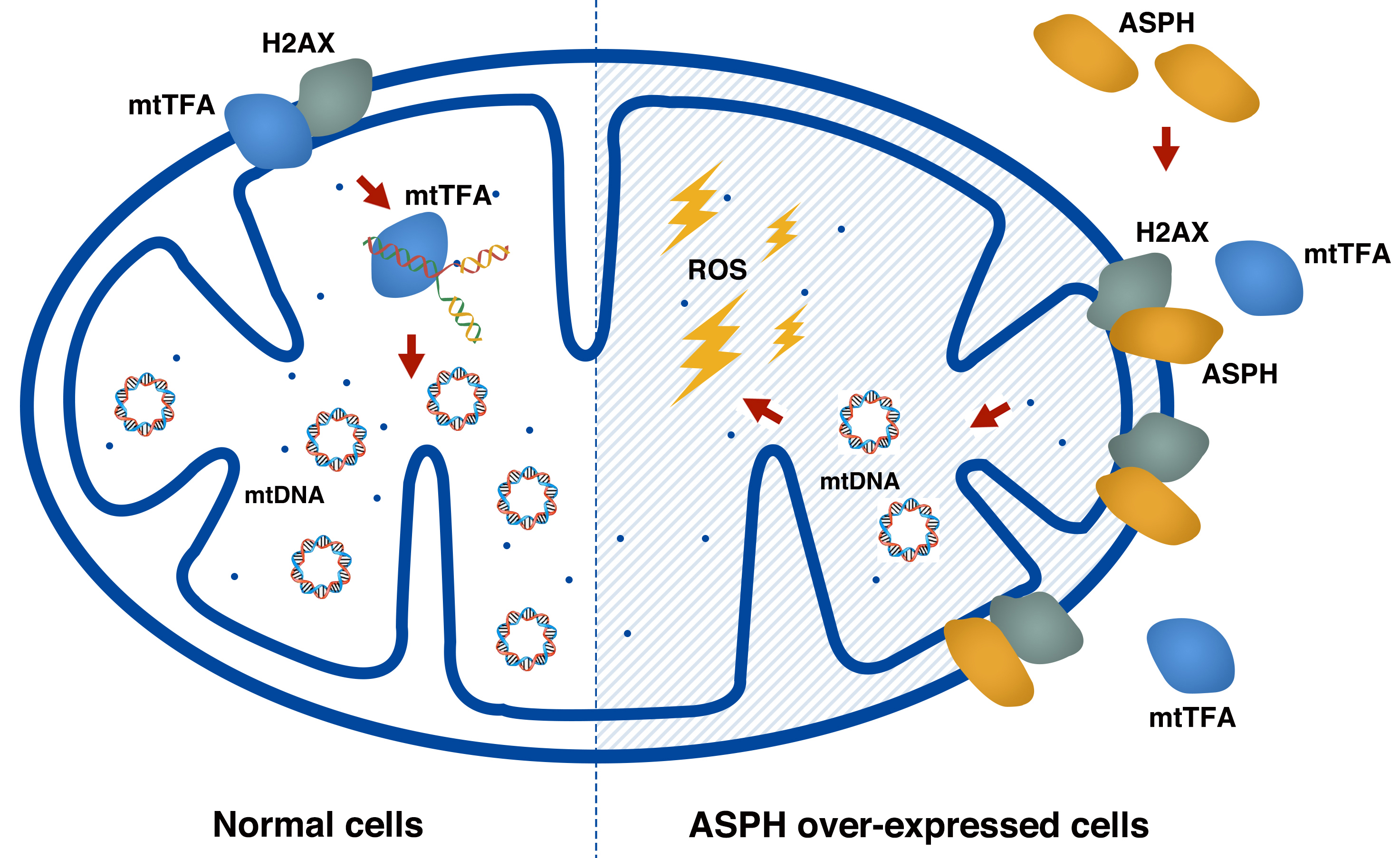
**

**Supplementary information, Figure S6. ASPH high or low expression in tumor tissues.**

(**a** and **b**) As previously reported,^25^ patients were grouped according to the level of ASPH expression. Specifically, the ratio of ASPH mRNA in the tumor tissues as compared with the matched non-tumor control tissues was tested by real-time PCR. Among 140 HCC patients, 34 patients had ASPH high expression (ratio > 2), while 37 patients had ASPH low expression in tumor tissues (ratio < 0.5). Lysates of tumor and matched non-tumor control tissues were immunoblotted by ASPH antibodies and actin antibodies. Actin served as a loading control. The representative results from each group were shown. T, tumor; N, matched non-tumor control.

**
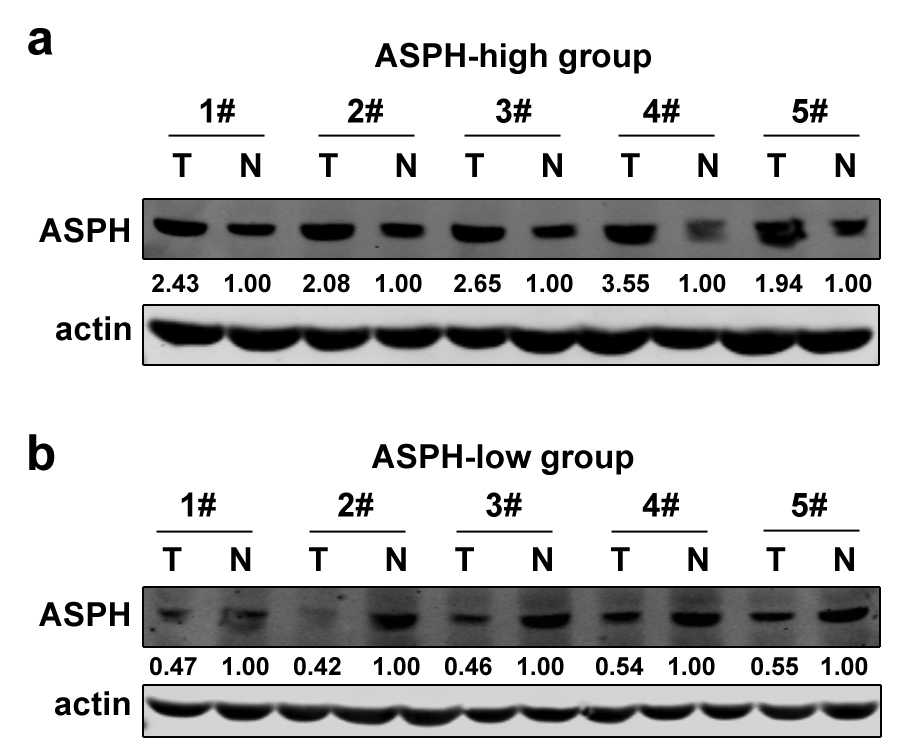
**

**Supplementary Tables**

**Supplementary information, Table S1**. **Clinicopathologic features in patients with high**

**or low ND-1 and D-loop copy numbers in HCC tissues**

| **Variable** | **Number (percent) / value (range)** | | |  | **Number (percent) / value (range)** | | |
| --- | --- | --- | --- | --- | --- | --- | --- |
|  | **ND1-low**  (n=44) | **ND1-high** (n=27) | **P value** |  | **D-loop-low** (n=48) | **D-loop-high** (n=23) | **P value** |
| **Age**, year | 52.9 (26.0-80.0) | 55.3 (37.0-71.0) | 0.283 |  | 54.3 (26.0-80.0) | 52.9 (36.0-69.0) | 0.353 |
| **Sex**, male | 38 (86.4%) | 23 (85.2%) | 0.890 |  | 41 (85.4%) | 20 (87.0%) | 0.861 |
| **HBsAg**, positive | 38 (86.4%) | 22 (81.5%) | 0.581 |  | 40 (83.3%) | 20 (87.0%) | 0.693 |
| **HBeAg**, positive | 13 (29.5%) | 8 (29.6%) | 0.994 |  | 13 (27.1%) | 8 (34.8%) | 0.506 |
| **Platelet**, 10^9^/L | 172.2 (56-387) | 142.9 (54-246) | 0.075 |  | 168.9 (56-387) | 144.6 (54-246) | 0.156 |
| **TBIL**, μmol/L | 14.0 (6.4-28.4) | 16.0 (7.0-36.5) | 0.161 |  | 14.0 (6.4-34.4) | 16.3 (7.0-36.5) | 0.108 |
| **ALB**, g/L | 42.2 (34.5-51.7) | 41.7 (35.7-47.3) | 0.568 |  | 42.0 (34.5-51.7) | 41.8 (35.7-47.3) | 0.808 |
| **ALT** U/L | 30.5 (8.0-242.0) | 40.0 (10.0-2530.0) | 0.122 |  | 33.0 (8.0-290.0) | 31.0 (10.0-2530.0) | 0.801 |
| **AFP**, μg/L, ≥20 | 32 (72.7%) | 13 (48.1%) | 0.037 |  | 31 (64.6%) | 14 (60.9%) | 0.761 |
| **Cirrhosis**, yes | 19 (43.2%) | 14 (51.9%) | 0.477 |  | 21 (43.8%) | 12 (52.2%) | 0.505 |
| **Tumor diameter**, cm, >5 | 32 (72.7%) | 15(55.6%) | 0.017 |  | 33 (68.7%) | 12 (52.2%) | 0.089 |
| **Tumor number**, multiple | 5 (11.4%) | 3 (11.1%) | 0.974 |  | 6 (12.5%) | 2 (8.7%) | 0.635 |
| **Tumor capsule**, incomplete | 25 (56.8%) | 10 (37.0%) | 0.106 |  | 28 (58.3%) | 7 (30.0%) | 0.028 |
| **PVTT**, yes | 11 (25.0%) | 3 (11.1%) | 0.153 |  | 11 (22.9%) | 3 (13.0%) | 0.328 |
| **MVI**, yes | 21 (47.7%) | 5 (18.5%) | 0.013 |  | 21 (43.8%) | 5 (21.7%) | 0.072 |
| **BCLC stage**, |  |  |  |  |  |  |  |
| 0-A | 11 (25.0%) | 14 (51.9%) | 0.058 |  | 13 (27.1%) | 12 (52.2%) | 0.114 |
| B | 22 (50.0%) | 10 (37.0%) |  |  | 24 (50.0%) | 8 (34.8%) |  |
| C | 11 (25.0%) | 3 (11.1%) |  |  | 11 (22.9%) | 3 (13.0%) |  |
| **TNM stage**, |  |  |  |  |  |  |  |
| I | 19 (43.2%) | 20 (74.1%) | 0.040 |  | 22 (45.8%) | 17 (74.0%) | 0.081 |
| II | 11 (25.0%) | 3 (11.1%) |  |  | 11 (22.9%) | 3 (13.0%) |  |
| III | 14 (31.8%) | 4 (14.8%) |  |  | 15 (31.3%) | 3 (13.0%) |  |

**Abbreviations:** ND-1, NADH dehydrogenase subunit 1; D-loop, displacement loop; HBsAg, hepatitis B surface antigen; HBeAg, hepatitis B e antigen;

TBIL, total bilirubin; ALB, albumin; ALT, alanine aminotransferase; AFP, alpha fetoprotein; PVTT, portal vein tumor thrombus; MVI, microvascular

invasion; BCLC, Barcelona Clinic Liver Cancer; TNM, tumor-node-metastasis.

**Supplementary information, Table S2**. **Clinicopathologic features in patients**

**with high or low ASPH expression in HCC tissues**

| **Variable** | **Number (percent) / value (range)** | | **P value** |
| --- | --- | --- | --- |
|  | **ASPH-low***  (n=37) | **ASPH-high***  (n=34) |  |
| **Age**, year | 53.4 (26.0-71.0) | 54.4 (34.0-80.0) | 0.703 |
| **Sex**, male | 31 (83.8%) | 30 (88.2%) | 0.590 |
| **HBsAg**, positive | 31 (83.8%) | 29 (85.3%) | 0.861 |
| **HBeAg**, positive | 12 (32.4%) | 9 (26.5%) | 0.582 |
| **Platelet**, 10^9^/L | 143.8 (56-255) | 178.9 (54-387) | 0.026 |
| **TBIL**, μmol/L | 14.6 (6.4-34.4) | 14.9 (7.0-36.5) | 0.818 |
| **ALB**, g/L | 41.7 (35.7-51.7) | 42.2 (34.5-49.1) | 0.573 |
| **ALT**, U/L | 34.0 (12.0-2530.0) | 28.5 (8.0-242.0) | 0.185 |
| **AFP**, μg/L, ≥20 | 21 (56.8%) | 24 (70.6%) | 0.227 |
| **Cirrhosis**, yes | 20 (54.1%) | 13 (38.2%) | 0.182 |
| **Tumor diameter**, cm, >5 | 18 (48.6%) | 26 (76.5%) | 0.016 |
| **Tumor number**, multiple | 3 (8.1%) | 5 (14.7%) | 0.380 |
| **Tumor capsule**, incomplete | 17 (45.9%) | 18 (52.9%) | 0.556 |
| **PVTT**, yes | 4 (10.8%) | 10 (29.4%) | 0.049 |
| **MVI**, yes | 13 (35.1%) | 13 (38.2%) | 0.786 |
| **BCLC stage**, |  |  |  |
| 0-A | 18 (48.7%) | 7 (20.6%) | 0.024 |
| B | 15 (40.5%) | 17 (50.0%) |  |
| C | 4 (10.8%) | 10 (29.4%) |  |
| **TNM stage**, |  |  |  |
| I | 22 (59.5%) | 17 (50.0%) | 0.036 |
| II | 10 (27.0%) | 4 (11.8%) |  |
| III | 5 (13.5%) | 13 (38.2%) |  |

**Abbreviations:** ASPH, aspartate β-hydroxylase; HBsAg, hepatitis B surface antigen; HBeAg, hepatitis B e antigen; TBIL, total bilirubin; ALB, albumin; ALT, alanine aminotransferase; AFP, alpha fetoprotein; PVTT, portal vein tumor thrombus; MVI, microvascular invasion; BCLC, Barcelona Clinic Liver Cancer; TNM, tumor-node-metastasis.

^*^ based on the results of RT-PCR.

**Supplementary information, Table S3. Sequences of PCR primers**

| **Gene** | **Forward Primers（5'-3'）** | **Reverse Primers（5'-3'）** |
| --- | --- | --- |
| ASPH mRNA | AAGGCGGACTCTCAGGAACT | AATCTCCATCACCATCAGCAT |
| β-actin mRNA | GATGACCCAGATCATGTTTGAG | AGGGCATACCCCTCGTAGAT |
| ND-1 | ATACAACTACGCAAAGGCCCCA | AATAGGAGGCCTAGGTTGAGGT |
| D-loop | TTGATTCCTGCCTCATCCTAT | GTCTGTGTGGAAAGTGGCTGT |
| β-actin DNA | CGGGAAATCGTGCGTGAC | GAAGGAAGGCTGGAAGAGTG |
| Full length D-loop | ACCAGTCTTGTAAACCGGAGAT | GATGCTTGCATGTGTAATCTTACTA |
| PXDN | AGGCAAGCATTTAAGGGACT | ACAGGATTTCACAGTCGCAGT |
| KRT1 | TCATTGACAAGGTGAGGTTCC | TTCCGAATCCAACCGAGAT |
| SQSTM1 | CGCAATGTTGGTTTCACTGA | TCTCTATGCAAACGTCATTGGT |
| GCLM | GTTGGGATACTGTGGGCTCT | AAGTAGATAATGTCGGCCCTG |
| PREX1 | AAGGCTACCTGTTGTCTCCG | TCTGCCGCTTGGTCTCAT |
| SOD3 | GAGCACTCAGAGCGCAAGA | GAGTGGAGGGTGTCTGTTGG |
| TPO | AAATACAGGCCCATCACAGG | GACTGAAGCCGTCCTCATAGA |
| MPO | ATCGGTTTTGGTGGGAGAA | CTTAGACACGGTGGTGATGC |
| mtTFA | CTCAAGTGATCCTTCCGAGTC | TTGATGCCAGGAGTTTGAGA |
| D-loop P1 | AACTAATACACCAGTCTTG | AATACATAGCGGTTGTTG |
| D-loop P2 | ACCGCTATGTATTTCGTA | TTGATGTGTGATAGTTGAG |
| D-loop P3 | CCACTAGGATACCAACAA | GTGCGGGATATTGATTTC |
| D-loop P4 | AATATCCCGCACAAGAGT | CCGTGAGTGGTTAATAGG |
| D-loop P5 | CATTTGGTATTTTCGTCTG | TAAGTATGTTCGCCTGTA |
| D-loop P6 | GGCGAACATACTTACTAA | AATTTGAAATCTGGTTAGG |
| D-loop P7 | AATTTTATCTTTTGGCGGTATG | TGTCTTTGGGGTTTGGTT |
| D-loop P8 | CCCCACAGTTTATGTAGC | TGCATGTGTAATCTTACTAAG |

**Abbreviations:** ASPH, aspartate β-hydroxylase; ND-1, NADH dehydrogenase subunit 1; D-loop, displacement loop; PXDN, peroxidasin homolog; KRT1, keratin 1; SQSTM1, sequestosome 1; GCLM, glutamate-cysteine ligase modifier subunit; PREX1, phosphatidylinositol-3,4,5-trisphosphate-dependent Rac exchange factor 1; SOD3, superoxide dismutase 3; TPO, thyroid peroxidase; MPO, myeloperoxidase; mtTFA, mitochondrial transcription factor A.

**Supplementary information, Table S4. The Human Oxidative Stress RT2 Profiler PCR Array results of HepG2 cells over-expressing ASPH, MHCC-97L cells with ASPH silenced and their respective controls.**

|  |  | **HepG2-ASPH** | **HepG2-con** | **MHCC-con** | **MHCC-siASPH** |
| --- | --- | --- | --- | --- | --- |
| **Well** | **Gene** | **Ct** | **Ct** | **Ct** | **Ct** |
| A1 | ALB | 33.000440 | 32.982803 | 23.517923 | 23.081848 |
| A2 | ALOX12 | 28.195028 | 29.432650 | 27.542694 | 28.240793 |
| A3 | AOX1 | 25.895203 | 25.720615 | 25.233519 | 25.840378 |
| A4 | APOE | 38.232900 | Undetermined | 34.709675 | 31.643644 |
| A5 | ATOX1 | 20.279003 | 19.956295 | 19.587198 | 20.104624 |
| A6 | BNIP3 | 19.702028 | 19.781210 | 19.585608 | 19.349460 |
| A7 | CAT | 20.701101 | 20.833510 | 20.658085 | 20.900250 |
| A8 | CCL5 | 26.200014 | 25.581995 | 27.532297 | 28.115713 |
| A9 | CCS | 23.014353 | 23.170881 | 22.720491 | 22.496914 |
| A10 | CYBB | 26.428976 | 27.139753 | 28.806654 | 27.371435 |
| A11 | CYGB | 29.057817 | 28.551617 | 29.456547 | 29.460182 |
| A12 | DHCR24 | 20.432133 | 20.653950 | 19.687477 | 21.734045 |
| B1 | DUOX1 | 28.311094 | 29.252970 | 26.744045 | 24.019611 |
| B2 | DUOX2 | 32.456050 | 32.390297 | 29.588478 | 25.977571 |
| B3 | DUSP1 | 16.450699 | 16.523039 | 20.798378 | 22.208443 |
| B4 | EPHX2 | Undetermined | Undetermined | 25.210686 | 25.613043 |
| B5 | EPX | 31.595755 | 31.474476 | 32.997543 | 32.718975 |
| B6 | FOXM1 | 19.102705 | 19.401823 | 19.114340 | 20.369318 |
| B7 | FTH1 | 12.968454 | 13.416118 | 12.918258 | 13.558314 |
| B8 | GCLC | 20.929811 | 21.283829 | 18.270514 | 19.491500 |
| B9 | GCLM | 23.276705 | 23.268917 | 17.162941 | 18.810637 |
| B10 | GPX1 | 16.946505 | 17.262344 | 18.002690 | 18.392096 |
| B11 | GPX2 | 27.697353 | 28.898030 | 17.286032 | 17.452332 |
| B12 | GPX3 | 28.961166 | 30.114399 | 22.161324 | 22.292755 |
| C1 | GPX4 | 18.681322 | 18.806221 | 17.216700 | 17.511530 |
| C2 | GPX5 | 33.153732 | 33.087517 | 32.867428 | 34.830470 |
| C3 | GPX6 | Undetermined | Undetermined | Undetermined | 31.738968 |
| C4 | GPX7 | 38.121464 | 32.303417 | 24.469595 | 23.488703 |
| C5 | GSR | 21.709930 | 22.006712 | 18.160873 | 19.691017 |
| C6 | GSS | 20.395947 | 20.493984 | 19.733435 | 20.781427 |
| C7 | GSTP1 | 17.414442 | 17.198221 | 17.702438 | 18.006105 |
| C8 | GSTZ1 | 21.529293 | 21.698107 | 21.934520 | 22.508947 |
| C9 | GTF2I | 19.782894 | 20.141455 | 18.953327 | 19.456423 |
| C10 | HMOX1 | 23.220367 | 23.643270 | 18.105011 | 18.702171 |
| C11 | HSPA1A | 19.586115 | 19.788822 | 18.312813 | 19.761150 |
| C12 | KRT1 | Undetermined | Undetermined | Undetermined | 33.436760 |
| D1 | LPO | Undetermined | 34.974575 | 39.538933 | 32.816765 |
| D2 | MB | 23.197115 | 23.445343 | 25.116676 | 25.465256 |
| D3 | MBL2 | Undetermined | 35.514170 | 27.171350 | 28.389948 |
| D4 | MGST3 | 17.735037 | 18.112780 | 18.341812 | 19.545180 |
| D5 | MPO | 32.974540 | Undetermined | 32.697304 | 32.123066 |
| D6 | MPV17 | 21.391184 | 21.418800 | 21.485748 | 21.888624 |
| D7 | MSRA | 23.308344 | 23.412415 | 23.128477 | 23.768633 |
| D8 | MT3 | Undetermined | Undetermined | Undetermined | Undetermined |
| D9 | NCF1 | Undetermined | Undetermined | Undetermined | Undetermined |
| D10 | NCF2 | 26.176369 | 26.264284 | 25.634634 | 27.319460 |
| D11 | NOS2 | 30.663038 | 30.638493 | 30.732733 | 30.370903 |
| D12 | NOX4 | 33.800224 | Undetermined | Undetermined | 34.814575 |
| E1 | NOX5 | 30.895449 | 32.173298 | 27.801310 | 27.776743 |
| E2 | NQO1 | 19.765532 | 19.892370 | 15.576063 | 16.348099 |
| E3 | NUDT1 | 21.872164 | 22.320082 | 21.161205 | 21.806358 |
| E4 | OXR1 | 23.320246 | 23.643969 | 21.905115 | 22.484549 |
| E5 | OXSR1 | 21.177206 | 21.179012 | 21.833319 | 22.390291 |
| E6 | PDLIM1 | 21.973272 | 20.965462 | 19.231327 | 19.966757 |
| E7 | PNKP | 26.789476 | 26.600613 | 25.802343 | 26.502686 |
| E8 | PRDX1 | 20.845705 | 21.148964 | 20.169752 | 21.037110 |
| E9 | PRDX2 | 18.102913 | 18.246930 | 19.558294 | 19.390550 |
| E10 | PRDX3 | 18.364740 | 18.390460 | 18.002886 | 18.871634 |
| E11 | PRDX4 | 18.211790 | 18.164942 | 19.885360 | 20.522877 |
| E12 | PRDX5 | 17.760664 | 17.776474 | 18.468845 | 18.886396 |
| F1 | PRDX6 | 17.523730 | 17.673030 | 17.990269 | 18.716482 |
| F2 | PREX1 | 26.393105 | 26.405098 | 23.463366 | 26.354366 |
| F3 | PRNP | 19.755041 | 19.818878 | 18.867850 | 20.229652 |
| F4 | PTGS1 | 30.679775 | 32.979065 | 26.248194 | 23.525629 |
| F5 | PTGS2 | 26.878735 | 27.013317 | 25.904154 | 24.804018 |
| F6 | PXDN | 22.283194 | 22.513264 | 38.215645 | 32.926346 |
| F7 | RNF7 | 20.130733 | 20.282830 | 19.695593 | 20.395900 |
| F8 | SCARA3 | 23.529211 | 23.458310 | 22.698872 | 23.422781 |
| F9 | SELS | 24.469915 | 24.529104 | 24.146778 | 25.323383 |
| F10 | SEPP1 | 27.689232 | 26.858780 | 27.219622 | 27.237673 |
| F11 | SFTPD | Undetermined | Undetermined | 30.460386 | 29.218960 |
| F12 | SIRT2 | 22.609453 | 22.949036 | 22.712751 | 23.284199 |
| G1 | SOD1 | 18.508293 | 18.819006 | 17.467869 | 18.345608 |
| G2 | SOD2 | 20.862741 | 21.690010 | 21.164540 | 22.137705 |
| G3 | SOD3 | Undetermined | 33.720078 | 25.178684 | 25.040224 |
| G4 | SQSTM1 | 17.399044 | 17.809841 | 14.317327 | 15.758367 |
| G5 | SRXN1 | 23.825575 | 23.670713 | 19.893055 | 22.142601 |
| G6 | STK25 | 21.800450 | 21.783173 | 21.782080 | 22.286186 |
| G7 | TPO | Undetermined | 34.011673 | Undetermined | 37.217323 |
| G8 | TTN | 38.626530 | 37.779423 | 28.915287 | 28.125480 |
| G9 | TXN | 18.862250 | 18.841671 | 15.421045 | 16.764580 |
| G10 | TXNRD1 | 19.524094 | 19.791773 | 16.051094 | 16.863064 |
| G11 | TXNRD2 | 24.203056 | 24.507055 | 23.208280 | 23.917600 |
| G12 | UCP2 | 24.589233 | 24.733215 | 24.114094 | 23.815903 |
| H1 | ACTB | 13.472939 | 13.586906 | 13.369909 | 14.654419 |
| H2 | B2M | 16.694185 | 17.022297 | 16.798807 | 17.676003 |
| H3 | GAPDH | 14.184049 | 14.583327 | 14.273161 | 14.161070 |
| H4 | HPRT1 | 20.883015 | 21.040915 | 20.356810 | 21.560266 |
| H5 | RPLP0 | 13.790972 | 13.948446 | 14.394459 | 15.437392 |
| H6 | HGDC | Undetermined | Undetermined | Undetermined | Undetermined |
| H7 | RTC | Undetermined | Undetermined | Undetermined | Undetermined |
| H8 | RTC | Undetermined | Undetermined | Undetermined | Undetermined |
| H9 | RTC | Undetermined | Undetermined | Undetermined | Undetermined |
| H10 | PPC | 18.853197 | 18.543365 | 18.748137 | 18.865170 |
| H11 | PPC | 18.680408 | 18.756128 | 18.696674 | 18.700432 |
| H12 | PPC | 19.134047 | 19.151289 | 19.159000 | 18.992940 |

**Abbreviations:** con, control; RTC, Reverse Transcription Control; PPC, Positive PCR Control.
